# A Universal Atomic Substitution Conversion Strategy Towards Synthesis of Large-Size Ultrathin Nonlayered Two-Dimensional Materials

**DOI:** 10.1007/s40820-021-00692-6

**Published:** 2021-08-05

**Authors:** Mei Zhao, Sijie Yang, Kenan Zhang, Lijie Zhang, Ping Chen, Sanjun Yang, Yang Zhao, Xiang Ding, Xiaotao Zu, Yuan Li, Yinghe Zhao, Liang Qiao, Tianyou Zhai

**Affiliations:** 1 School of Physics, University of Electronic Science and Technology of China (UESTC), Chengdu, 610054 People’s Republic of China; 2 State Key Laboratory of Materials Processing and Die & Mould Technology, School of Materials Science and Engineering, Huazhong University of Science and Technology (HUST), Wuhan, 430074 People’s Republic of China; 3 School of Materials and Energy, Guangdong University of Technology, Guangzhou, 510006 People’s Republic of China; 4 Key Laboratory of Carbon Materials of Zhejiang Province, Institute of New Materials and Industrial Technologies, College of Chemistry and Materials Engineering, Wenzhou University, Wenzhou, 325035 People’s Republic of China

**Keywords:** Nonlayered 2D materials, Large-size ultrathin CdS flakes, Atomic substitution conversion, Layered-nonlayered structural transformation

## Abstract

Nonlayered two-dimensional (2D) materials have attracted increasing attention, due to novel physical properties, unique surface structure, and high compatibility with microfabrication technique. However, owing to the inherent strong covalent bonds, the direct synthesis of 2D planar structure from nonlayered materials, especially for the realization of large-size ultrathin 2D nonlayered materials, is still a huge challenge. Here, a general atomic substitution conversion strategy is proposed to synthesize large-size, ultrathin nonlayered 2D materials. Taking nonlayered CdS as a typical example, large-size ultrathin nonlayered CdS single-crystalline flakes are successfully achieved via a facile low-temperature chemical sulfurization method, where pre-grown layered CdI_2_ flakes are employed as the precursor via a simple hot plate assisted vertical vapor deposition method. The size and thickness of CdS flakes can be controlled by the CdI_2_ precursor. The growth mechanism is ascribed to the chemical substitution reaction from I to S atoms between CdI_2_ and CdS, which has been evidenced by experiments and theoretical calculations. The atomic substitution conversion strategy demonstrates that the existing 2D layered materials can serve as the precursor for difficult-to-synthesize nonlayered 2D materials, providing a bridge between layered and nonlayered materials, meanwhile realizing the fabrication of large-size ultrathin nonlayered 2D materials.

**Supplementary Information:**

The online version contains supplementary material available at 10.1007/s40820-021-00692-6.

## Highlights

- A general layered 2D materials-derived atomic substitution conversion strategy is proposed to achieve the synthesis of large-size ultrathin nonlayered 2D materials.
- Using low-melting-point CdI_2_ flakes via a simple hot plate assisted vertical vapor deposition method as precursor, large-size ultrathin CdS flakes were successfully converted from layered to nonlayered nanostructures through a facile low-temperature chemical sulfurization process.
- The size and thickness of CdS flakes can be controlled by the CdI_2_ precursor. The growth mechanism is ascribed to the chemical substitution reaction from I to S atoms between CdI_2_ and CdS, which has been evidenced by experiments and theoretical calculations.

**Supplementary Information:**

The online version contains supplementary material available at 10.1007/s40820-021-00692-6.

## Graphical abstract

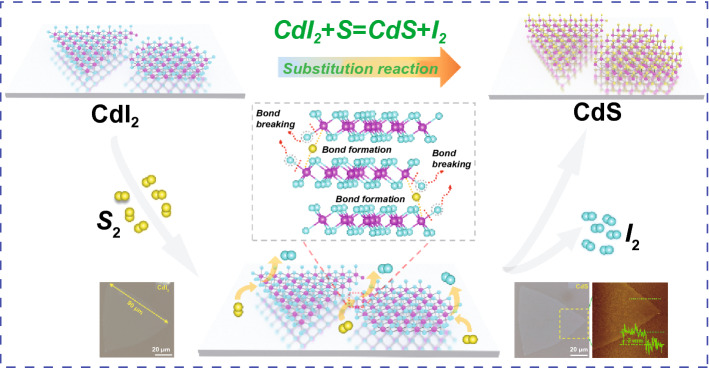

## Introduction

The two-dimensional (2D) materials with atomically thin thickness, including layered and nonlayered materials, have gained extensive research interest in recent years, owing to their abundant species, diverse exotic properties, broad promising applications [1–6]. Particularly, nonlayered 2D materials formed by strongly intrinsic covalent bonds in three-dimensional (3D) directions without van der Waals (vdW) gap, occupy the majority among the tens of thousands of 2D materials, in contrast to layered 2D materials with strong in-plane chemical bonding and naturally weak interplane vdW interaction [1, 2, 7]. Moreover, in comparison with layered 2D materials, nonlayered 2D materials possess both the novel features of their bulks and 2D characteristics inducing abundant distinct phenomena necessary complement to layered 2D materials [8, 9]; nonlayered 2D materials own large lattice structural distortion in the 2D limit absent in layered materials, leading to massive number of surface dangling bonds and further greatly enhancing the surface chemical activity [8, 10]; nonlayered 2D materials also have strong mechanical flexibility favoring high compatibility with traditional Si substrate and integration capability with current microfabrication techniques [11, 12]. Thus, nonlayered 2D materials have attracted dramatical attention for various promising applications in modern electronics, optoelectronics, catalysis, energy storage and conversion [13–19].

However, it remains greatly difficult to prepare nonlayered 2D materials at present, in contrast to layered 2D materials. Specially, due to the intrinsically strong 3D bonding character, the nonlayered 2D materials are unable to suspend stably at 2D scale within Born–Oppenheimer surface consideration, resulting in tendency to stack into 3D nanostructures induced by surface energy constraints [5, 20–22]. Moreover, the creation of 2D morphology from nonlayered 2D materials usually requires to introduce additional driving force to overcome the intrinsic surface tension and stabilize the crystal structure [8, 10, 20]. Currently, to promote 2D anisotropic growth of nonlayered materials, three main synthetic strategies have been adopted to obtain 2D planar structure of nonlayered materials, namely external force-assisted lamellar exfoliation strategy [23, 24], vdW substrate-guided anisotropic growth strategy [25–28], synergistic additive-mediated growth strategy [29–31]. The external force-assisted lamellar exfoliation strategy usually suffers from small lateral size, irregular morphology and random distribution of the products [23, 24]; vdW epitaxy substrate-guided anisotropic growth strategy usually requires harsh preparation conditions such as relative high growth temperature [25–28]; synergistic additive-mediated growth strategy would inevitably introduce foreign elements, degrading the crystal quality [29–31]. In general, current synthetic strategies cannot provide a satisfying solution for large-size ultrathin nonlayered 2D materials with high quality. Therefore, it is especially important and highly urgent to develop effective routes to synthesize nonlayered 2D materials with desired morphology and quality, in order to realize their practical industry applications in future.

Herein, a general layered 2D materials-derived atomic substitution conversion strategy is proposed to achieve the synthesis of large-size ultrathin nonlayered 2D materials. In this work, taking CdS as a typical nonlayered 2D material with stable wurtzite structure for example, using low-melting-point CdI_2_ flakes via a simple hot plate assisted vertical vapor deposition (HPVVD) method as precursor, large-size ultrathin CdS flakes were successfully converted from layered to nonlayered nanostructures through a facile low-temperature chemical sulfurization process. The converted CdS flakes demonstrate large-size (up to submillimeter scale), ultrathin thickness (down to 2 nm), high-quality single crystals. Specially, the lateral size and vertical thickness of CdS flakes can be controlled by the CdI_2_ flakes precursor. The conversion mechanism was revealed via experiments and theoretical calculations, which might be attributed to an atomic-level chemical substitution reaction from I to S atoms between layered CdI_2_ and nonlayered CdS. The presented generalized atomic substitution conversion strategy may open a new avenue to synthesize ultrathin nonlayered 2D materials with large size.

## Experimental Section

### Growth of Layered CdI_2_ Flakes

2D CdI_2_ flakes were prepared by a facile HPVVD method in ambient environment. First, trace amount of CdI_2_ powder precursor was transferred on a clean glass slide and then placed on a hot plate at room temperature. Meanwhile, two glass slides with thickness of 1 mm were mounted on both sides of the precursor glass slide. The temperature of the hot plate was then ramped to expected growth temperature (360–400 °C), and a freshly cleaved mica substrate was put on top of the precursor. 2D CdI_2_ flakes started to deposit on the mica substrate immediately and the mica substrate can be removed soon. The whole fabrication process took only a few minutes under atmospheric pressure. The CdI_2_ flakes can also grow on SiO_2_/Si and quartz substrates other than mica substrate.

### Conversion of Layered CdI_2_ into Nonlayered CdS Flakes

The conversion from CdI_2_ to CdS flakes was conducted in an atmospheric pressure chemical vapor deposition (APCVD) furnace system. In a typical process, 300 mg S powder was placed at the center heating zone of the tubular furnace, and the substrates with as-grown CdI_2_ flakes were placed 3 to 8 cm on the downstream side. The evaporation temperature of S powder at the central zone was increased to 280–300 °C and maintained for 30–60 min. The whole conversion process was achieved at atmospheric pressure with 100 sccm Ar used as carrier gas. Finally, the furnace was naturally cooled down to room temperature.

### Characterization of CdI_2_ and CdS Flakes

The surface morphology, domain size and thickness of CdI_2_ and CdS crystals were characterized by optical microscope (OM) (OLYMPUS, BX51) and atomic force microscopy (AFM) (Bruker, Dimension Icon). The crystal structure, phase, and composition were analyzed using X-ray powder diffraction (XRD) (Bruker, D2 phaser), X-ray photoelectron spectroscopy (XPS) (Kratos, AXIS-ULTRA DLD-600 W), microscope-based Raman spectrometer (WITec, Alpha 300RS+, 532 nm excitation laser) equipped with a 100X objective lens, and transmission electron microscopy (TEM) (FEI, Tecnai G2 F30) equipped with an energy-dispersive X-ray spectroscopy (EDS) system.

### Fabrication and Performance Measurement of CdS Devices

The photodetector based on CdS flakes was fabricated through a previously reported transfer electrode method [32, 33]. Briefly, Au electrodes were firstly deposited on a hydrophobic SiO_2_/Si substrate by the thermal evaporation machine (Nexdep, Angstrom Engineering), and then picked up carefully assisted by the liquid Ga metal, and finally transferred to the desired CdS flake. The optoelectrical properties were measured on a probe station (Lakeshore, TTPX) which was linked to a semiconductor device analyzer (Keysight, B1500A) and a 365 nm laser with an adjustable optical attenuator. Notably, all the photoelectrical measurements are conducted at room temperature in ambient condition.

### Theory Calculations

The thermodynamic function and data were calculated from the HSC chemistry 6. The density functional theory (DFT) calculations were performed in the Vienna Ab initio Software Package (VASP) [34]. The projected augmented wave (PAW) method and the Perdew–Burke–Ernzerhof of generalized gradient approximation (PBE-GGA) were employed [35–37]. A plane-wave energy cutoff of 500 eV was adopted. The *k*-point of CdI_2_ and CdS was set as 1 × 1 × 1. 0.01 eV Å^−1^ and 10^–5^ eV was used as the maximum energy difference and residual forces, respectively.

## Results and Discussion

### Conversion Process of Layered CdI_2_ Flakes into Nonlayered CdS Flakes

**Fig. 1:**
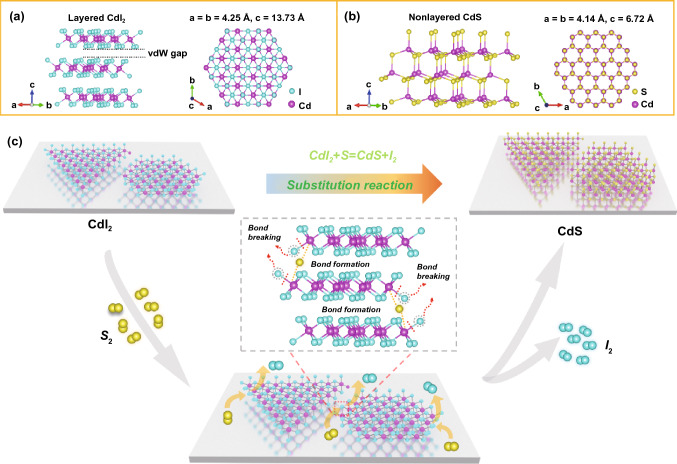
Schematic illustration and synthesis process of the conversion in crystal structure from layered CdI_2_ to nonlayered CdS via chemical substitution reaction by addition of S powder. **a**, **b** Crystal structure of layered CdI_2_ (hexagonal) and nonlayered CdS (hexagonal), respectively. **c** Conversion process of CdI_2_ to CdS through substitution of I atoms by S atoms

Figure 1 schematically illustrates the synthesis process of nonlayered CdS flakes by conversion from layered CdI_2_ flakes as precursor via vapor-phase chemical sulfurization treatment. As shown in Fig. 1a, b, CdI_2_ has a typical layered structure with a hexagonal unit cell, and crystallizes in space group *P*63*mc* (186) with lattice constants of *a* = *b* = 4.25 Å, *c* = 13.73 Å; whereas CdS is a representative nonlayered material with a hexagonal unit cell, and belongs to space group *P*63*mc* (186) with lattice constants of *a* = *b* = 4.14 Å, *c* = 6.72 Å. Note that CdI_2_ and CdS have similar crystal symmetry, which is an important factor to promote the conversion process. We also need to point out while CdI_2_ is a typically layered material with weak vdW force, CdS has a nonlayered crystal structure without vdW gap, which is significant meaningful for layered-nonlayered structural conversion to form nonlayered crystal structure. Figure 1c displays the conversion process from CdI_2_ to CdS through a simple sulfurization reaction. In detail, the S atoms replace the I atoms by exposing CdI_2_ under S-rich vapors accompanied by the breaking of Cd-I bonds and the formation of Cd-S bonds, resulting in the successful conversion from layered to nonlayered structural transformation. It should be noted that the as-grown CdI_2_ flakes were firstly synthesized as the growth precursor for transferring into CdS flakes via a facile HPVVD method in ambient condition, followed by the chemical sulfurization process to form CdS flakes (see Experimental Section for details). Importantly, the HPVVD is actually a simple and efficient vertical vapor deposition process, which is a totally open heating system conducted in a mild atmospheric environment; this novel method avoids expensive equipment and harsh reaction conditions (e. g., high vacuum environment, high growth temperature and long growth time), compared to traditional physical/chemical vapor deposition (PVD/CVD) technology [38–42]. More importantly, owning to the low melting point of both the S (112 °C) and CdI_2_ (404 °C), the chemical sulfurization process can be performed at relatively low temperature (280–300 °C) compared to common CVD, resulting in the direct conversion from metal halides to chalcogenides without obvious change in 2D planar shape and size.

### Growth and Characterization of Layered CdI_2_ Flakes

**Fig. 2:**
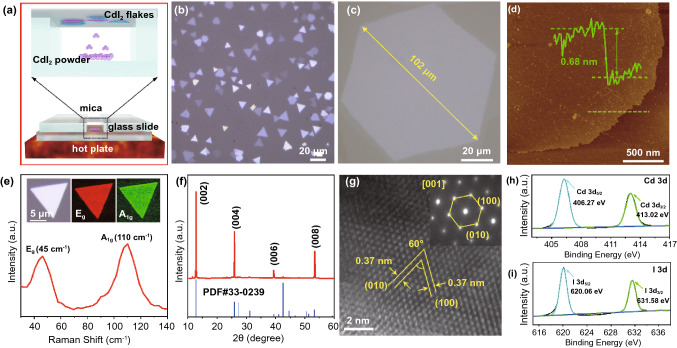
Growth and characterization of layered CdI_2_ flakes. **a** Schematic drawing of HPVVD setup for growth of CdI_2_ flakes on mica substrate. **b** The typical OM image of large-area CdI_2_ flakes grown on mica substrate showing both triangular and hexagonal geometry. **c** Representative OM image of a large-size CdI_2_ flake with hexagonal shape on mica substrate. **d** AFM image and corresponding height profile of a typical monolayer CdI_2_. **e** Raman spectrum of CdI_2_ flakes. The inset is the typical OM and Raman intensity mapping (E_g_ and A_1g_ mode) of a triangular CdI_2_ flake. **f** XRD pattern of CdI_2_ flakes grown on SiO_2_/Si substrate. **g** HRTEM image of CdI_2_ flakes. The inset is the SAED pattern taken along the direction [001] of CdI_2_ flakes. **h**, **i** XPS spectra of Cd 3d and I 3d peaks from CdI_2_ flakes

Firstly, large-scale ultrathin CdI_2_ single-crystal flakes were grown by a facile and efficient HPVVD technique. Figure 2a presents the HPVVD apparatus, which consists merely of a hot plate and several glass slides as glass mounting bracket to achieve microspacing between the CdI_2_ powder precursor and the mica substrate. The whole growth procedure can be totally accomplished under ambient pressure in air within only a few minutes, which is simple and quick devoid of any rigorous experimental conditions as required by conventional vapor deposition implemented in a horizontally placed quartz tube. Simply adjusting growth temperature (360–400 °C), growth time (1–2 min) and spacing distance (1–2 mm) between the precursor and the substrate, which are considered as key growth factors in such vertical deposition configuration, abundant CdI_2_ flakes with different thickness are fully grown on the mica substrate, displaying regular triangular or hexagonal shape with smooth surface and sharp edges, as shown in Fig. 2b. The 2D flake-like CdI_2_ crystals are featured with well-defined morphology determined by the intrinsic hexagonal crystal structure, indicating the single-crystalline nature. Notably, the maximum lateral size of CdI_2_ flakes can even surpass 100 μm (Fig. 2c), which is much larger than traditional PVD-based CdI_2_ flakes reported so far [43]. Moreover, the thickness of ultrathin CdI_2_ flakes can be thinned down to about 0.68 nm (Fig. 2d), which corresponds to the theoretical thickness of monolayer CdI_2_ consisting of only one unit [I-Cd-I].

Furthermore, a series of characterizations and analysis of the as-grown CdI_2_ flakes were conducted to verify the crystal structure and chemical composition. Figure 2e presents the typical Raman spectra of CdI_2_ flakes grown on mica substrate. Obviously, there are two prominent and strong characteristic peaks around 45 and 110 cm^−1^, which correspond to the in-plane (E_g_) and the out-of-plane (A_1g_) phonon vibration mode, respectively, in consist with the recently reported 2D CdI_2_ crystals [43, 44]. The inset is the representative Raman intensity maps of E_g_ and A_1g_ modes of a typical CdI_2_ triangular flake, indicating highly uniform crystalline quality throughout the entire flake. Figure 2f shows the X-ray diffraction (XRD) patterns of CdI_2_ flakes grown on SiO_2_/Si substrate specially (in order to avoid the interference of mica substrate). It is clear that four sharp XRD peaks (12.88°, 25.92°, 39.37°, and 53.35°) can all be well indexed to {001} family planes ((002), (004), (006), (008)) of standard hexagonal phase CdI_2_ (PDF#33–0239), implying the (001) plane preferred growth orientation and good crystalline quality of as-synthesized CdI_2_ flakes. To further elucidate the crystal orientation and microstructure, TEM analysis was performed to characterize CdI_2_ flakes, as exhibited in Fig. 2g. The high-resolution TEM (HRTEM) image and the corresponding selected area electron diffraction (SAED) pattern display clearly resolved lattice fringes and a single set of sharp diffraction spots in hexagonal symmetry, further confirming the high single-crystalline nature of the CdI_2_ flakes. The measured lattice spacing along two different directions with 60° interfacial angle is 0.37 nm, corresponding to the {110} family planes of hexagonal phase CdI_2_. The results of HRTEM and SAED indicate that the (001) plane is the preferred growth orientation of CdI_2_ flakes, in accordance with the standard XRD pattern of hexagonal phase of CdI_2_. In addition, the elemental composition of as-prepared CdI_2_ samples was identified by the XPS, as depicted in Fig. 2h, i. The two strong peaks at around 406.27 and 413.02 eV are attributed to Cd 3d_5/2_ and Cd 3d_3/2_, respectively, meanwhile the peaks locating at 620.06 and 631.58 eV are assigned to I 3d_5/2_ and I 3d_3/2_, respectively. All above results prove that the as-grown CdI_2_ flakes are high-quality hexagonal single-crystal structure composed of (001) plane as preferential growth orientation. Hence, large-size ultrathin CdI_2_ flakes with high single-crystalline quality were successfully synthesized by a simple and effective HPVVD method, which is the basis of subsequent conversion from CdI_2_ flakes to obtain large-size ultrathin nonlayered CdS flakes.

### Conversion Process of Layered CdI_2_ Flakes into Nonlayered CdS Flakes

**Fig. 3:**
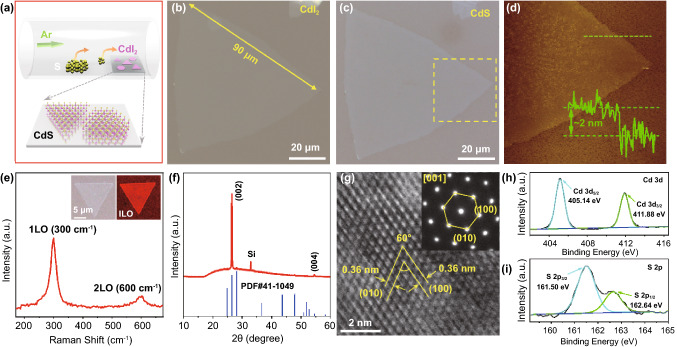
Growth and characterization of nonlayered CdS flakes. **a** Schematic presentation of conversion setup and atomic structure of CdS flakes on mica substrate by chemical sulfurization method. **b**, **c** Representative OM images of original large-scale CdI_2_ thin flake and converted CdS thin flake on mica substrates, respectively. **d** AFM image and corresponding height profile of thin CdS flake in panel c. **e** Raman spectrum of converted CdS flakes. The inset is the typical OM and Raman intensity mapping (1LO mode) of the triangular CdS flake. **f** XRD pattern of converted CdS flakes on SiO_2_/Si substrate. **g** HRTEM image of converted CdS flakes. The inset is the SAED pattern taken along the direction [001] of CdS flakes. **h**, **i** XPS spectra of Cd 3d and S 2p peaks from converted CdS flakes

Subsequently, large-size ultrathin CdS single-crystalline flakes were successfully converted from HPVVD-grown CdI_2_ flakes via low-temperature chemical sulfurization process. Figure 3a is a schematic of experimental setup for the sulfurization process, where the Ar gas transports vaporized S to the heated CdI_2_ flakes on mica substrate. Briefly, the conversion reaction was carried out in a quartz tube with Ar gas as carrier gas. The sulfur powder was placed at the center of the tubular furnace at 280–300 °C, while the pre-grown CdI_2_ flakes were placed at the downstream of the furnace. The Ar gas transports the vaporized sulfur to the heated CdI_2_ flakes to realize the conversion which depends on the chemical reaction between CdI_2_ flakes and chemically reactive sulfur vapor to form CdS flakes. It should be noted that the growth/transition temperature of the chemical sulfurization process is relatively low in contrast to the most nonlayered 2D materials [25, 29, 45]. Figure 3b, c exhibits the typical OM images for the original CdI_2_ flake with submillimeter size and the corresponding converted CdS flake, respectively. As can be seen, there is no obvious change in surface topography and lateral size of the resulting CdS flake as compared with the pristine CdI_2_ flake after chemical sulfurization. Figure 3d presents the corresponding AFM image for the converted CdS flake (marked in square region in Fig. 3c) with the ultrathin thickness (~ 2 nm), displaying relatively atomically sharp edges with a clean and smooth surface. The corresponding dark-field OM image also indicates uniform surface and clear domain boundary of the converted CdS flake, as shown in Fig. S1. Note that the lateral length is the largest size, and the thickness is the smallest thickness of 2D CdS flakes reported so far [29, 46–49]. Besides, large-area CdS flakes with various thickness were also successfully converted from parent CdI_2_ flakes (Fig. S2). Therefore, owning to the precursor-directed synthesis method, the resulting CdS flakes inherit all advantages of the parent CdI_2_ flakes, containing well-defined 2D lamellar structure and a high yield.

The Raman spectra collected from the converted CdS flakes are shown in Fig. 3e. It is found that the previously established CdI_2_ Raman peaks at 45 and 110 cm^−1^ disappear, while new peaks emerge at 300 and 600 cm^−1^, which can be determined as the first and second-order longitudinal optical (1LO and 2LO) phonon modes of CdS phase, respectively, in consistent with the previously reported PVD-based CdS flakes [29, 50]. The strong and uniform Raman intensity over the entire CdS flake (the inset of Figs. 3e and S3) indicates that the resulting products still maintain high uniformity and complete conversion of CdI_2_ into CdS. The crystalline structure of converted CdS samples was studied by XRD (Fig. 3f). Note that the XRD patterns of the resulting CdS flakes obtained from in situ conversion from the as-grown CdI_2_ flakes on SiO_2_/Si substrate (Fig. 2f). It is clear that two new peaks (26.51° and 53.35°) have appeared, which can be indexed to (002) and (004) crystalline planes of standard hexagonal CdS pattern (PDF#41-1049) without the observation of secondary phase, suggesting phase purity of the CdS products and (001) plane preferred exposed facet after the conversion process. It is worth noting that the diffraction peaks of CdI_2_ disappeared entirely, indicating a complete conversion of layered CdI_2_ into nonlayered CdS crystals. TEM was utilized to further explore the atomic structure of converted CdS crystals, as shown in Fig. 3g. The HRTEM image reveals relatively clear lattice fringes with interplanar spacing of (100) and (010) planes for hexagonal structure of CdS crystals. The SAED pattern presents only a set of clear and sharp hexagonal diffraction spots, which are well indexed to (100) and (010) planes and thus (001) plane is identified as the preferential exposed facet, in line with the XRD results, together verifying the excellent crystallinity quality of the resulting CdS flakes. Moreover, XPS analysis was performed to affirm the elemental composition of converted CdS products (Fig. 3h, i). It is apparent that the new peaks emerging at 161.50 and 162.24 eV, corresponding to S 2p_3/2_ and S 2p_1/2_, respectively, whereas the peaks (405.14 and 411.88 eV) are attributed to Cd 3d_5/2_ and Cd 3d_3/2_, respectively, further confirming the successful conversion into CdS crystals. Together, as discussed above, large-size ultrathin nonlayered CdS flakes with excellent crystallization were successfully and completely converted from CdI_2_ flakes as precursor through the facile low-temperature chemical sulfurization process. Furthermore, based on the same atomic substitution conversion strategy, nonlayered materials CdS (layered CdBr_2_ as the 2D precursor) and CdSe (layered CdI_2_ as the 2D precursor) have been successfully synthesized confirmed by both Raman spectroscopy and XRD characterizations (Fig. S4) [31, 51–53], demonstrating the universality of this novel strategy.

### Growth Mechanism of CdS Flakes

**Fig. 4:**
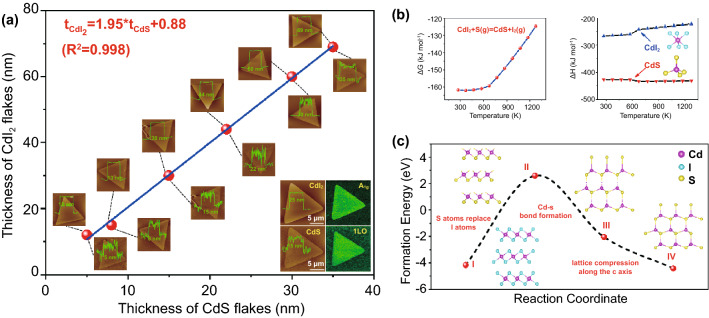
The controllable sulfidation of CdI_2_ flakes and corresponding conversion mechanism of CdS flakes. **a** The thickness of CdI_2_ flakes before (AFM images above data line) and after being converted to CdS flakes (AFM images below data line). Inset: the representative AFM images and corresponding Raman intensity mapping of CdI_2_ flakes and converted CdS flakes. Note that the thickness of the CdI_2_ flakes was about 1.95 times higher compared to the corresponding of CdS flakes, which agrees well with the ratio of the c lattice constant between the two compounds. **b**, **c** The theory calculations and the proposed growth mechanism of CdS flakes. **b** Left: the calculated Gibbs free energy changes versus the temperature for the chemical reaction concerning the conversion process. Right: the calculated formation enthalpy changes versus the temperature for CdI_2_ and CdS. **c** The DFT-calculated energy profile of the possible reaction pathway for CdS

To understand the underlying conversion process from layered CdI_2_ flakes to nonlayered CdS flakes in-depth, both experiments and theoretical calculations were performed to explore the growth/transformation mechanism. Firstly, the topography and thickness of 2D CdI_2_ flakes before and after being converted to CdS flakes were systematically investigated by AFM, as shown in Fig. 4a. It is clearly observed that the morphology (including surface shape and lateral dimension) of converted CdS flakes is almost identical to that of the original CdI_2_ flakes, probably reflecting the similar hexagonal crystal structure of CdI_2_ and CdS. Interestingly, the thickness of pristine CdI_2_ flakes and converted CdS flakes (before and after sulfurization conversion process) correlates to each other by 1.95, where the thickness of original CdI_2_ flakes was about 1.95 times higher than that of the corresponding converted CdS flakes, in good agreement with the ratio of the lattice constant between the two compounds along the c axis (*C*_CdI2_/*C*_CdS_ = 13.73/6.72 Å = 2.04). Moreover, the AFM and the corresponding Raman intensity mapping characterizations were further employed to verify the complete conversion of CdI_2_ into CdS, as demonstrated in the inset of Fig. 4a. This can provide an effective route to control the morphology and thickness of the corresponding nonlayered 2D materials.

Furthermore, the theory calculations were performed to clarify the conversion mechanism of chemical transformation from CdI_2_ to CdS at atomic scale on the basis of the morphology retaining and thickness reducing phenomena observed in experiments, as depicted in detail in Fig. 4b, c). From the aspect of thermodynamics, the conversion process occurs between CdI_2_ and S vapor supplied by heating S powder when the temperature reach 280–300 °C, following the chemical reaction: CdI_2_ + S (g) = CdS + I_2_ (g). As shown in Fig. 4b, the calculated Gibbs free energy changes (∆G) are always negative in 273.15–1273.15 K, suggesting the conversion process is spontaneous sulfurization substitution reaction, in which ∆G = ∆H − *T*∆S, where ∆H, T, and ∆S is formation enthalpy changes, Kelvin temperature, and entropy changes, respectively. Moreover, the calculated formation enthalpy changes of CdS are always much negative than CdI_2_ in 273.15–1273.15 K, indicating CdS is more stable than CdI_2_ during the conversion process, namely Cd atoms prefer to form chemical bonding with S atoms rather than I atoms. Besides, the possible reaction pathway was proposed to interpret the growth process based on the atomic chemical substitution reaction from CdI_2_ to CdS, and the corresponding DFT-simulated energy profile was provided to investigate the crystal structure transformation from layered CdI_2_ to nonlayered CdS when the S atoms substitute I atoms, as depicted in detail in Fig. 4c. The potential atomic growth mechanism might undergo the chemical compound transformation of I (CdI_2_), II (transition state of CdS), III (transition state of CdS), and IV (CdS). Hence, the mechanism could be simply divided into three-step process, including S atoms replacing I atoms, Cd-S bond formation, and lattice compression along the c axis of CdS. Initially, owing to the volatility of I atoms and high local partial gas pressure of S_2_ molecular at high temperature, S atoms tend to substitute I atoms along the in-plane of CdI_2_, yielding an intermediate state with energetically unfavorable structure (II). Subsequently, to maintain more stable structure (III), the adjacent Cd layers are bridged by the S atoms gradually, meanwhile keeping the same lattice constant with CdI_2_ along the c axis, resulting in the disappearing of the vdW gaps that originally exist in layered CdI_2_. Finally, new Cd-S bonds form along the out-of-plane through lattice compression of c axis, contributing to form thermodynamically stable CdS (IV) product by comparison with the calculated formation energy of four chemical compounds. It is worth pointing out that layered CdI_2_ (*a* = *b* = 4.25 Å) and nonlayered CdS (*a* = *b* = 4.14 Å) have similar hexagonal crystal structure in the lateral direction (along the *a* and *b* axis direction of CdI_2_ and CdS). It means that breaking Cd-I bonds and forming Cd-S bonds do not need large rearrangements of Cd atoms, although small migration of positions of Cd atoms might be necessary to compensate the bond length difference between Cd-I and Cd-S, thus allowing gentle transformation without crystal structural collapse in the lateral direction, and leading to the morphology maintaining before and after chemical conversion. Whereas in the vertical direction (along the c axis direction of CdI_2_ and CdS), the distance between two Cd layers in CdI_2_ is 6.84 Å, while it is 3.42 Å in CdS without vdW gaps, determined by the intrinsic crystal structures of them. Thus, the thickness of the flake can be decreased to 50% after the chemical transforming from CdI_2_ to CdS in the theory.

### Optoelectronic Performance of CdS Flakes

**Fig. 5:**
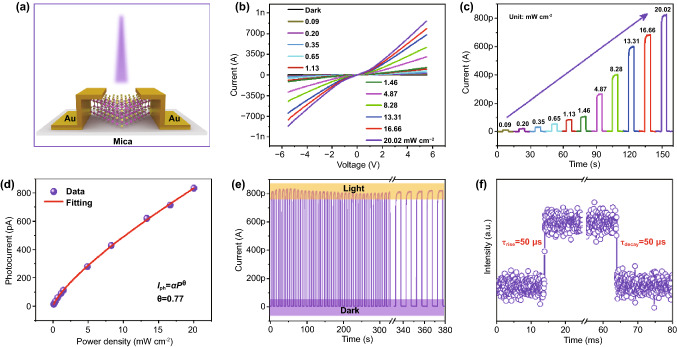
Optoelectronic performance of a converted CdS flake-based photodetector. **a** The schematic view of the photodetector based on CdS flake on mica substrate. **b** The *I*–*V* curves of the device in the dark and under 365 nm laser illumination with various light intensity. **c** The corresponding light power density-dependent photoresponse under 365 nm laser illumination at *V*_bias_ = 5 V. **d** The corresponding fitting curve of photocurrent versus power density. **e**, **f** The photoresponse to on/off laser irradiation show (**e**) the stability (**f**) the typical response rate of the device

To examine the optoelectronic properties of the converted CdS flakes, the photodetector was in situ constructed on individual CdS flake on mica substrate via transfer electrode method (see Experimental Section for details). The schematic diagram of a typical converted CdS flake-based photodetector under 365 nm laser illumination is presented in Fig. 5a. The corresponding OM image and AFM height profile (the thickness is ~ 20 nm) are given in Fig. S5. Figure 5b shows the current versus voltage (*I-V*) characteristic curves of the device in the dark and under 365 nm laser illumination with a series of different light intensities. The corresponding time-dependent photoresponse is exhibited in Fig. 5c. Clearly, a pronounced photoconductive response is observed under laser illumination, indicating the outstanding photoresponse of the device which results from the enhancement of photo-generated carriers. Specially, this converted 2D CdS flake-based photodetector displays an ultralow dark current (*I*_dark_: ~ 120 fA) even at *V*_bias_ = 5 V, reflecting the high-resistance characteristics of CdS flakes. Thus, the corresponding photoswitching ratio (*I*_ph_/*I*_dark_) can reach up to 10^3^ (6942) under 20.02 mW cm^−2^ (*V*_bias_ = 5 V), highly comparable and even superior to PVD-grown CdS flake-based and many other typical 2D nonlayered materials-based photodetectors [25–29, 45, 53, 54], verifying remarkable photoresponse to the ultraviolet light. Moreover, the power density-dependent photoresponse could be fitted as a power law, *I*_ph_ = *αP*^θ^, where *α*, *θ,* and *P* represent the constant for a certain wavelength, power factor, and power density of incident light, respectively. As plotted in Fig. 5d, the fitting *θ* is 0.77, indicating such sublinear behavior possibly caused by trap states in converted CdS flakes, which was also observed in PVD-grown CdS flakes [29]. Furthermore, as crucial performance parameters of the photodetector, the responsivity (*R*) and the specific detectivity (D*), can be expressed as *R* = *I*_ph_/*PS*, and D* = *RS*^1/2^/(2*eI*_dark_)^1/2^), respectively, where *I*_ph_, *S*, and *e* represent photocurrent (*I*_ph_ = *I*_light_ -*I*_dark_), the effective illuminated area between channels, and elementary electronic charge, respectively. Hence, the obtained *R*_max_ is 56.48 mA W^−1^, and the corresponding D* is 4.53 × 10^8^ Jones under 0.09 mW cm^−2^ at 5 V bias. In addition, to further access the optoelectronic performance of the converted CdS flake-based photodetector, the stability and response speed were explored by switching the laser on and off. Figure 5e displays the time-dependent photoresponse of the device with the laser on/off time interval of 10 s at *V*_bias_ = 5 V, which demonstrates almost the same level of dark current and photocurrent after multiple cycles in 380 s hold time, suggesting highly stable and repeatable photoresponse to the light. More importantly, as key parameters to evaluate the crystalline quality of materials, the rise time (*τ*_rise_) and decay time (*τ*_decay_), defined as the needed time of current variation between 10% and 90% of the maximum current, were calculated to be about 50 μs, which is much lower than PVD-grown CdS flakes and most nonlayered 2D materials [25–29, 45, 53, 54], indicating ultrafast photoresponse speed deriving from the high-crystalline quality of the converted CdS flakes. Overall, the photodetector based on the converted CdS flakes displays ultralow dark current, ultrahigh current on/off ratio, and ultrafast photoresponse speed, whose superior photoresponse performances are primarily ascribed to high-quality single-crystalline feature of the converted CdS flakes, further confirming the effectivity of the atomic substitution conversion strategy.

## Conclusions

In summary, large-size ultrathin nonlayered CdS flakes were successfully synthesized by a facile atomic substitution conversion strategy, which transform from layered CdI_2_ flakes as the precursor via a simple HPVVD method. The resulting CdS flakes are high-quality singe crystals with the lateral size up to submillimeter scale and the thickness down to atomic layers level. Besides, the morphology, size and thickness of CdS flakes were well controlled by the precursor of CdI_2_ flakes. The atomic substitution conversion mechanism was confirmed by both of experiments and theoretical calculations, which could be attributed to the chemical substitution reaction from I to S atoms between CdI_2_ and CdS, meanwhile realizing in situ structural transformation from layered to nonlayered 2D materials. More importantly, this novel atomic substitution conversion strategy can be extended to the fabrication of other nonlayered 2D materials like CdSe. The proposed general pre-grown layered 2D precursor-directed atomic substitution conversion strategy may offer a new route to prepare large-size ultrathin nonlayered 2D materials.

## Supplementary Information

Below is the link to the electronic supplementary material.

Supplementary file1 (PDF 479 kb)
