# Supplementary material for: A Universal Atomic Substitution Conversion Strategy Towards Synthesis of Large-Size Ultrathin Nonlayered Two-Dimensional Materials
Source: Nanomicro Lett. 2021 Aug 5;13:165. doi: 10.1007/s40820-021-00692-6 (PMC8342677; doi:10.1007/s40820-021-00692-6)
Supplement: Supplementary file 1 — Supplementary file1 (PDF 479 kb) [file 40820_2021_692_MOESM1_ESM.pdf]

Supporting Information for

A Universal Atomic Substitution Conversion Strategy Towards Synthesis of Large-Size Ultrathin Nonlayered Two-Dimensional Materials

Mei Zhao[1, 2], Sijie Yang[2], Kenan Zhang[3], Lijie Zhang[4], Ping Chen[2], Sanjun Yang[2], Yang Zhao[1], Xiang Ding[1], Xiaotao Zu[1], Yuan Li[2], Yinghe Zhao[2], Liang Qiao[1, *], Tianyou Zhai[2, *]

[1]School of Physics, University of Electronic Science and Technology of China (UESTC), Chengdu 610054, P. R. China

[2]State Key Laboratory of Materials Processing and Die & Mould Technology, School of Materials Science and Engineering, Huazhong University of Science and Technology (HUST), Wuhan 430074, P. R. China

[3]School of Materials and Energy, Guangdong University of Technology, Guangzhou 510006, P. R. China

[4]Key Laboratory of Carbon Materials of Zhejiang Province, Institute of New Materials and Industrial Technologies, College of Chemistry and Materials Engineering, Wenzhou University, Wenzhou 325035, P. R. China

*Corresponding authors. E-mail: liang.qiao@uestc.edu.cn (Liang Qiao); zhaity@hust.edu.cn (Tianyou Zhai)

Supplementary Figures

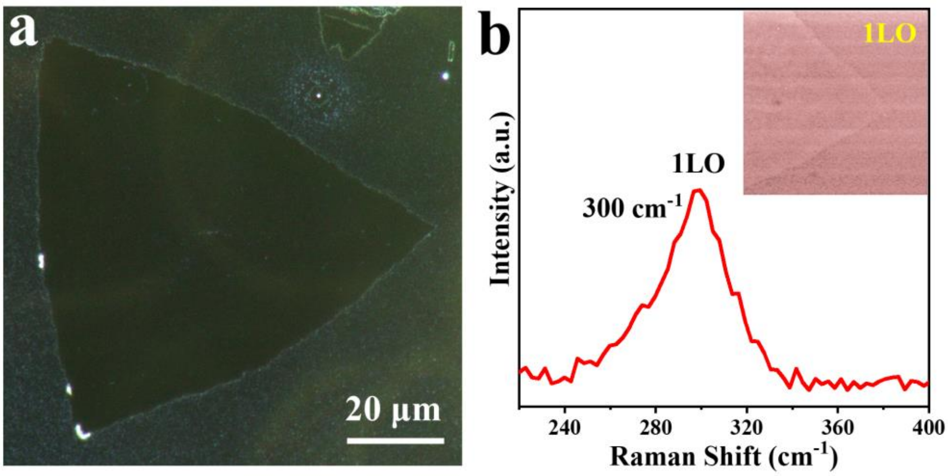

Fig. S1 Dark-field OM image (**a**) and Raman spectrum (**b**) of large-size ultrathin CdS flake in Fig. 3c

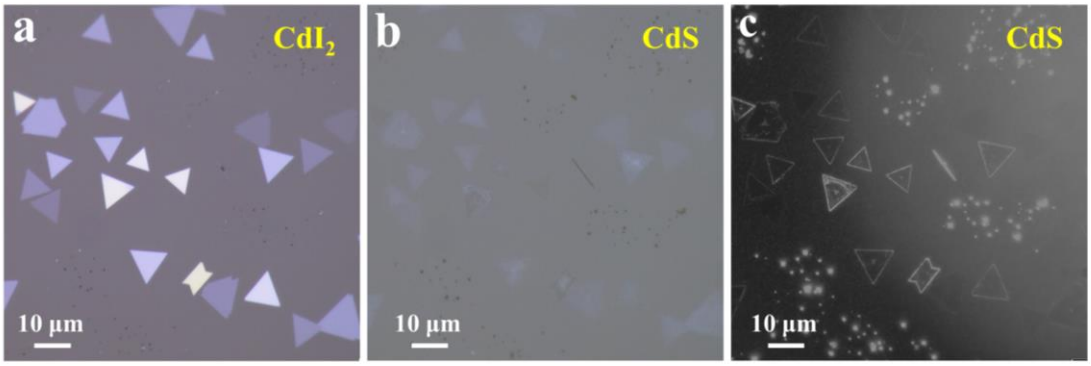

Fig. S2 Typical bright-field and dark-field OM images of large-area CdI_2 flakes with various thickness before (**a**) and after (**b, c**) conversion into CdS flakes

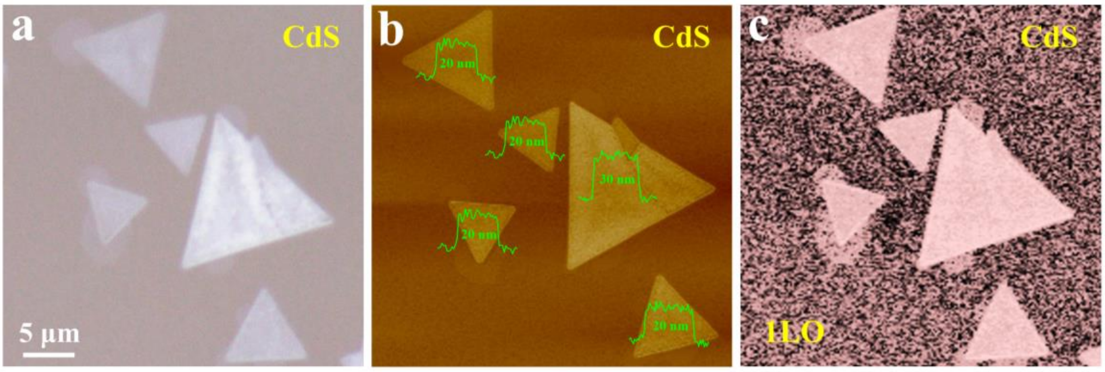

Fig. S3 Typical OM image (**a**) and corresponding AFM image (**b**), and Raman intensity mapping of converted CdS flakes with different thickness (**c**)

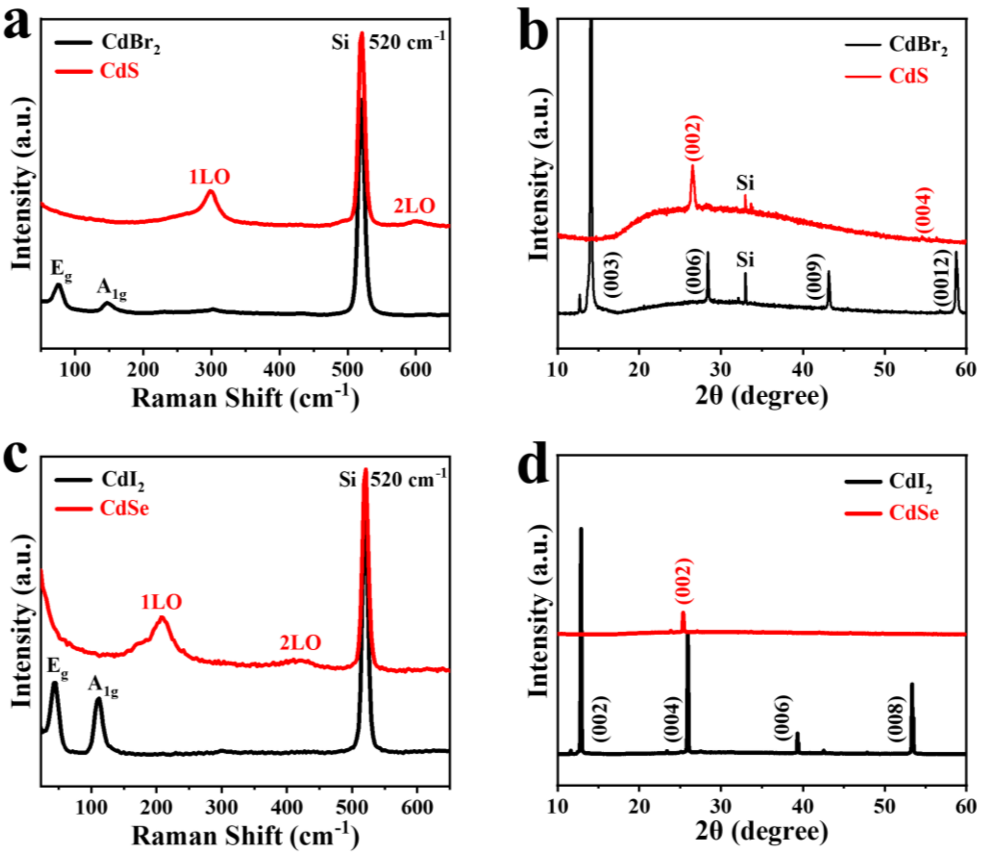

Fig. S4 a Raman spectra of $CdBr_2$ flakes grown on SiO_2/Si substrate and corresponding converted CdS flakes. **b** XRD pattern of $CdBr_2$ flakes grown on SiO_2/Si substrate and corresponding converted CdS flakes. **c** Raman spectra of CdI_2 flakes grown on SiO_2/Si substrate and corresponding converted CdSe flakes. **d** XRD pattern of CdI_2 flakes grown on SiO_2/Si substrate and corresponding converted CdSe flakes

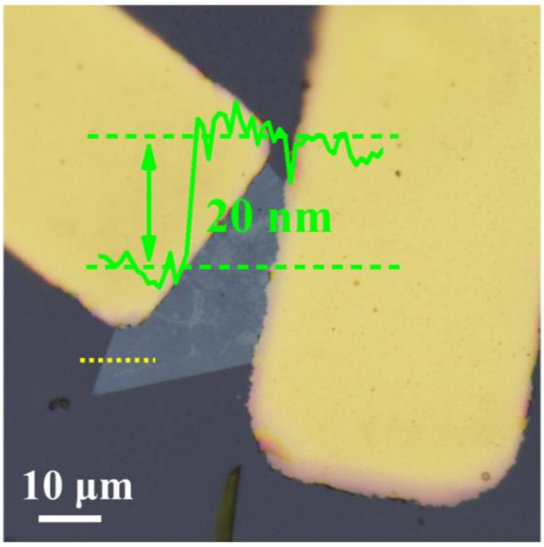

Fig. S5 OM image and corresponding AFM height profile of the converted CdS flake-based photodetector via the transfer electrode method
